# Supplementary material for: Integrating geospatial tools in mapping forest fire severity and burned areas in the Western Usambara Mountain Forests, Lushoto, Tanzania
Source: PLoS One. 2025 Jun 6;20(6):e0311865. doi: 10.1371/journal.pone.0311865 (PMC12143507; doi:10.1371/journal.pone.0311865)
Supplement: Attachment — Submitted filename: Response to Reviewers.docx [file pone.0311865.s002.docx]

**Response to Journal Academic editor and reviewer**

**Journal Requirements:**

1. Please ensure that your manuscript meets PLOS ONE's style requirements, including those for file naming. The PLOS ONE style templates can be found at
   https://journals.plos.org/plosone/s/file?id=wjVg/PLOSOne_formatting_sample_main_body.pdf and 
   <https://journals.plos.org/plosone/s/file?id=ba62/PLOSOne_formatting_sample_title_authors_affiliations.pdf>

- *We have made every effort to adhere to the Journal’s requirements and standards, including the specific guidelines for file naming. The revised manuscript has been formatted in accordance with these standards.*

2. You indicated that ethical approval was not necessary for your study. We understand that the framework for ethical oversight requirements for studies of this type may differ depending on the setting and we would appreciate some further clarification regarding your research. Could you please provide further details on why your study is exempt from the need for approval and confirmation from your institutional review board or research ethics committee (e.g., in the form of a letter or email correspondence) that ethics review was not necessary for this study? Please include a copy of the correspondence as an ""Other"" file.

- *Thank you, Editor, for highlighting the importance of ethical approval for this study. Initially, it was determined that ethical approval was not necessary; however, upon further assessment, the* ***Directorate of Postgraduate Studies, Research, Technology Transfer, and Consultancy (DPRTC)*** *at* ***Sokoine University of Agriculture*** *approved the study as is in line with research ethics. This decision was made because the research involves human subjects through questionnaires designed to gather information on forest fire sources and effects. Therefore, this document is included and attached as an “Others” file*

3. We note that the grant information you provided in the ‘Funding Information’ and ‘Financial Disclosure’ sections do not match. When you resubmit, please ensure that you provide the correct grant numbers for the awards you received for your study in the ‘Funding Information’ section.

- *Thank you, Editor, for this feedback. We have revised the funding information and Financial Disclosure sections to ensure that the grant number accurately matches the award received for our study. We appreciate your guidance on this matter for further improvement of the manuscript.* *Currently, the funding statement reads as follows:*

*"B.P.Mkiwa - Name of the author received the award*

*Grant number: 2022/RP/M000710160*

*Name: Tanzania Forest Fund (TaFF)*

*TaFF* [*URL:https://www.mfukowamisitu.go.tz*](URL:https://www.mfukowamisitu.go.tz)

*The sponsor or funder did not play any role in the study design, data collection and analysis, decision to publish, or manuscript preparation”.*

4. Thank you for stating the following in the Competing Interests section:
   " I have read the journal's policy and the authors of this manuscript have the following competing interests: Reports of financial support provided by the Tanzania Forest Services Agency (TFS) and Tanzania Forest Fund (TaFF)." Please confirm that this does not alter your adherence to all PLOS ONE policies on sharing data and materials, by including the following statement: "This does not alter our adherence to PLOS ONE policies on sharing data and materials.” (as detailed online in our guide for authors http://journals.plos.org/plosone/s/competing-interests). If there are restrictions on sharing of data and/or materials, please state these. Please note that we cannot proceed with consideration of your article until this information has been declared.  Please include your updated Competing Interests statement in your cover letter; we will change the online submission form on your behalf.

- *Thank you for your guidance regarding the competing interest’s section. We have revised this section and made it more correct with minor changes and should read as follows: “I* have read the journal's policy and the authors of this manuscript have the following competing interests: Reports of financial support provided by the Tanzania Forest Fund (TaFF)." *I confirm the statement, “This does not alter our adherence to PLOS ONE policy on sharing data materials”. Furthermore, there are no restrictions on sharing data and materials related to this study when needed.*

5. Thank you for stating the following in the Acknowledgments Section of your manuscript:
   "The authors thank the Tanzania Forest Services Agency (TFS) and Tanzania Forest Fund (TaFF) for financial support for field work and manuscript development. “We note that you have provided funding information that is currently declared in your Funding Statement. However, funding information should not appear in the Acknowledgments section or other areas of your manuscript. We will only publish funding information present in the Funding Statement section of the online submission form. 
   Please remove any funding-related text from the manuscript and let us know how you would like to update your Funding Statement.

- *This section has been revised in the manuscript by removing the funding information and replacing it with acknowledgment details. Additionally, the funding statement has been updated on the award Grant number. Currently, the funding statement reads as follows:*

*"B.P. Mkiwa - Name of the author who received the award*

*Grant number: 2022/RP/M000710160*

*Name: Tanzania Forest Fund (TaFF)*

*TaFF* [*URL:https://www.mfukowamisitu.go.tz*](URL:https://www.mfukowamisitu.go.tz)

*The sponsor or funder did not play any role in the study design, data collection and analysis, decision to publish, or manuscript preparation”. These amendments I request to be included in the online submitted form.*

6. We note that your Data Availability Statement is currently as follows: All relevant data are within the manuscript and its Supporting Information files. Please confirm at this time whether or not your submission contains all raw data required to replicate the results of your study. Authors must share the “minimal data set” for their submission. PLOS defines the minimal data set to consist of the data required to replicate all study findings reported in the article, as well as related metadata and methods (https://journals.plos.org/plosone/s/data-availability#loc-minimal-data-set-definition).For example, authors should submit the following data:

- The values behind the means, standard deviations, and other measures reported;
- The values used to build graphs;
- The points extracted from images for analysis.

Authors do not need to submit their entire data set if only a portion of the data was used in the reported study.

a) If your submission does not contain these data, please either upload them as Supporting Information files or deposit them to a stable, public repository and provide us with the relevant URLs, DOIs, or accession numbers. For a list of recommended repositories, please see <https://journals.plos.org/plosone/s/recommended-repositories>.
b) If there are ethical or legal restrictions on sharing a de-identified data set, please explain them in detail (e.g., data contain potentially sensitive information, data are owned by a third-party organization, etc.) and who has imposed them (e.g., an ethics committee). Please also provide contact information for a data access committee, ethics committee, or other institutional body to which data requests may be sent. If data are owned by a third party, please indicate how others may request data access.

- *All relevant data are included within the manuscript; I confirm that my submission contains all raw data required to replicate the results of this study.* *However, some raw data are available free of charge to all users on the GEE platform and can be obtained here:* [*https://code.earthengine.google.com/*](https://code.earthengine.google.com/)*. Otherwise, can be available from the corresponding author upon reasonable request.*

7. Please include your full ethics statement in the ‘Methods’ section of your manuscript file. In your statement, please include the full name of the IRB or ethics committee who approved or waived your study, as well as whether or not you obtained informed written or verbal consent. If consent was waived for your study, please include this information in your statement as well.

- *This research was conducted following the ethical standards approved by the* ***Directorate of Postgraduate Studies, Research, Technology Transfer, and Consultancy (DPRTC) of Sokoine University of Agriculture (SUA)****, under approval reference number:* ***DPRTC/R/186/35****. Informed consent was obtained from all participants before their involvement in the study. All procedures involving human subjects were designated to ensure confidentiality and minimize any potential risks. Participants were fully informed about the purpose of the study, which focuses on forest fire sources, effects, and management aspects while respecting their rights to withdraw at any time without penalty. During the interviews, we obtained informed written and verbal consent from participants. Approval letters have been included for your consideration, and this information has been included in the methods section of the manuscript and the cover letter.*

8. We note that Figures 1 and 7 in your submission contain map/satellite images which may be copyrighted. All PLOS content is published under the Creative Commons Attribution License (CC BY 4.0), which means that the manuscript, images, and Supporting Information files will be freely available online, and any third party is permitted to access, download, copy, distribute, and use these materials in any way, even commercially, with proper attribution. For these reasons, we cannot publish previously copyrighted maps or satellite images created using proprietary data, such as Google software (Google Maps, Street View, and Earth). For more information, see our copyright guidelines: http://journals.plos.org/plosone/s/licenses-and-copyright.
    
   We require you to either
i. Present written permission from the copyright holder to publish these figures specifically under the CC BY 4.0 license, or
ii. remove the figures from your submission:

- You may seek permission from the original copyright holder of Figures 1 and 7 to publish the content specifically under the CC BY 4.0 license.  
   
  We recommend that you contact the original copyright holder with the Content Permission Form (http://journals.plos.org/plosone/s/file?id=7c09/content-permission-form.pdf) and the following text:
  “I request permission for the open-access journal PLOS ONE to publish XXX under the Creative Commons Attribution License (CCAL) CC BY 4.0 (http://creativecommons.org/licenses/by/4.0/). Please be aware that this license allows unrestricted use and distribution, even commercially, by third parties. Please reply and provide explicit written permission to publish XXX under a CC BY license and complete the attached form.”
   
  Please upload the completed Content Permission Form or other proof of granted permissions as an ""Other"" file with your submission. In the figure caption of the copyrighted figure, please include the following text: “Reprinted from [ref] under a CC BY license, with permission from [name of publisher], original copyright [original copyright year].”
- If you are unable to obtain permission from the original copyright holder to publish these figures under the CC BY 4.0 license or if the copyright holder’s requirements are incompatible with the CC BY 4.0 license, please either i) remove the figure or ii) supply a replacement figure that complies with the CC BY 4.0 license. Please check copyright information on all replacement figures and update the figure caption with source information. If applicable, please specify in the figure caption text when a figure is similar but not identical to the original image and is therefore for illustrative purposes only.
  The following resources for replacing copyrighted map figures may be helpful:
   
  USGS National Map Viewer (public domain): http://viewer.nationalmap.gov/viewer/
  The Gateway to Astronaut Photography of Earth (public domain): http://eol.jsc.nasa.gov/sseop/clickmap/
  Maps at the CIA (public domain): https://www.cia.gov/library/publications/the-world-factbook/index.html and https://www.cia.gov/library/publications/cia-maps-publications/index.html
  NASA Earth Observatory (public domain): http://earthobservatory.nasa.gov/
  Landsat: http://landsat.visibleearth.nasa.gov/
  USGS EROS (Earth Resources Observatory and Science (EROS) Center) (public domain): http://eros.usgs.gov/#
  Natural Earth (public domain): <http://www.naturalearthdata.com/>
  - **Figure 1 and 7** utilizes forest shapefiles obtained from the World Database on Protected Areas (WDPA). According to the instructions for data usage, it is required to properly credit the source. The citation format provided is:
    *IUCN and UNEP-WCMC (year), The World Database on Protected Areas (WDPA) [Online], [insert month/year of the version downloaded], Cambridge, UK: UNEP. Available at:* [*www.protectedplanet.net*](http://www.protectedplanet.net)*.*
  - *The satellite image background for the study area map was generated using QGIS, a free and open-source geographic information system (GIS). QGIS enables users to create, edit, visualize, analyze, and publish geospatial information without requiring a license. The map utilized the Planet Explorer plugin, an open-source QGIS plugin that provides access to PlanetScope imagery and base maps.*
  - *Planet offers a free basic subscription to students, researchers, and staff at accredited universities. Additionally, through its partnership with Norway's International Climate and Forest Initiative (NICFI), Planet provides free access to high-resolution satellite imagery, including PlanetScope data, for various users in Tanzania and other regions.*
  - *The NICFI program aims to reduce and reverse tropical forest loss, combat climate change, conserve biodiversity, and promote sustainable development. The satellite data provided under this initiative is widely used by governments, civil society, the private sector, and academia to advance the NICFI goals of forest conservation, biodiversity protection, and forest regrowth.*
  - *Figure 7 illustrates the analyzed Landsat 8 and 9 imageries obtained from the United States Geological Survey (USGS) via Google Earth Engine. Landsat imagery, produced by the U.S. federal government, serves various purposes, with a primary focus on Earth observation and vegetation monitoring.*
  - *As stated in the usage guidelines: "When using any imagery, data products, or information accessed from USGS or NASA’s LP DAAC systems, proper credit must be given. The proper use and citation of imagery and products will vary according to source, and detailed information is provided in the sections below."*
  - *To acknowledge the USGS as the data source, a citation line should be included, such as:*

*Landsat-8 and 9 images courtesy of the U.S. Geological Survey*

**Reviewer #1 critical comments**

1. **Abstract**
a) Clarity and Conciseness: The abstract should clearly summarize the key objectives, methods, findings, and implications in a more concise manner. Currently, it may feel too detailed for an abstract.
   - *We have further revised the abstract to present all sections more concisely (Summarized key objectives, methods, findings, and the implication of the study)*
b) Quantitative Highlights: Include specific quantitative results, such as the percentage of areas affected by fires, in the abstract to provide immediate insight into the study's significance.
   - *Thank you for your valuable insight, we have included the percentage of the area affected by fire in our revisions.*
c) Implications: Briefly mention the practical implications of the findings in the abstract to highlight their relevance to stakeholders.
   - *Thank you for your valuable insight, we have revised and included in the manuscript that “Practically These findings serve as a reminder for policymakers to develop targeted strategies for reducing forest fire risks, while community engagement and education can promote sustainable practices and enhance awareness for fire management.*
2. **Introduction**
a) Literature Review: The introduction should include a more comprehensive literature review on forest fires in Tanzania and the use of geospatial tools. This contextualizes the study within existing research and highlights gaps that the study addresses.
   - *The manuscript has been enhanced with a more comprehensive literature review on forest fires in Tanzania, emphasizing the role of geospatial tools and the significance of community involvement in forest fire management. This includes a focus on ensuring that community rights are respected in the management process.*
b) Research Questions: Clearly state the specific research questions or hypotheses at the end of the introduction to guide the reader on what to expect.
   - *The research questions in the manuscript have been revised to better guide the reader’s expectations regarding the study.*
c) Local Context: Provide more background on the socio-economic context of the Western Usambara Mountain area, including the local communities' reliance on forest resources, which will strengthen the rationale for the study.
   - *The manuscript has been revised by providing more background on all mentioned aspects. It now includes detailed information on how anthropogenic activities, particularly those conducted by the community, are contributing to the increasing problem of forest fires in the Usambara Mountains areas.*
3. **Methodology**
a) Method Justification: Explain why specific geospatial tools and methodologies (e.g., dNBR, dNDVI) were chosen over other potential methods. This can enhance the credibility of the approach.
   - *The use of dNBR and dNDVI for classifying forest fires is favored over other methods due to their sensitivity and effectiveness in detecting burn severity, soil conditions, and vegetation loss before and after fire, allowing for precise detection of burn severity. dNBR offers a continuous scale for assessing fire impacts, while dNDVI focuses on vegetation health, making them complementary tools for understanding ecological effects. Both indices have been validated against field data, enhancing their reliability for monitoring fire effects and supporting resource management efforts. Their versatility allows for application across various ecosystems, facilitating the identification of areas needing rehabilitation and informing strategies to mitigate future fire risks. Additionally, their effectiveness is particularly notable in mountainous forest areas like West Usambara Forest where Magamba Nature Forest Reserve, Mkusu Forest Reserve, and Shagayu Forest Reserve study areas are located and where traditional methods may be less applicable.*
b) Sampling Details: Clarify the sampling strategy for the Participatory Rural Appraisal (PRA) and how participants were selected. This will help assess the representativeness of the data.
   - *In this study, the Participatory Rural Appraisal (PRA) sampling strategy involved purposive sampling. Two villages were selected from three pre-identified forest reserves, specifically chosen based on fire incidence, distance from the forest, and residence. Respondents were then randomly determined from community members directly involved in forest use, Use of fire management, or fire handling. This approach ensured that the perspectives of those most affected by and knowledgeable about forest fires were adequately represented in the study.*
c) Temporal Analysis: Provide more detail on the temporal resolution of satellite imagery used and how it aligns with fire events. This is crucial for understanding changes over time.
   - *With a temporal resolution of 16 days, Landsat 8 and 9 provide consistent and reliable observations for tracking forest fires over time. This frequency is beneficial for post-fire analysis and long-term monitoring because it allows systematic tracking of changes in burn severity, vegetation recovery, and regrowth trends. Landsat imagery captures initial burn severity and vegetation damage shortly after a fire, and subsequent revisit intervals provide crucial data for evaluating restoration efforts and natural recovery. The 16-day interval creates a temporal archive that supports the detection of gradual changes and anomalies, as well as the comparison of fire events across several years to understand seasonal or climatic trends. Additionally, the integration of thermal bands in Landsat 8 and 9 improves the analysis of land cover. Landsat 8 and 9 are crucial instruments for efficient forest fire management and decision-making because they combine temporal and spectral capabilities.*
d) Limitations: Discuss any potential limitations in the methodologies used, such as uncertainties in remote sensing data or challenges in ground validation.

- *Thank you for this important oversight. In fact, using this method has some limitations including;*

i. *Remote sensing requires ground truthing to validate findings, but in some areas like in the Western Usambara Mountain Forest, it is very difficult to validate satellite observations since the areas are characterized by elevated mountains.*
ii. Resolution limitation when capturing small or medium-sized fires which can either be under-estimated or over-estimated in the extent of burn areas and misclassify burn severity
iii. *Both are effective when assessing areas shortly after the fire, but as vegetation begins to regenerate, the spectral signals change leading to potential misclassifications of burn severity, therefore distinguishing between burned and recovering areas becomes challenging.*
iv. *In the tropics both the NBR and NDVI are less effective due to quick changes in vegetation regrowth and quick changes in reflectance properties which can hinder the detection of fire impacts.*
4. **Results**
a) Data Presentation: Use clearer visuals, such as graphs or maps, to present key findings. This can enhance comprehension and highlight important trends more effectively.

- *Thank you for your feedback; the figures and tables have been revised for improved clarity and visual representation*

b) Statistical Analysis: Provide more detailed statistical analyses to support claims about correlations or trends observed in the data.

***The relationship between NDVI and NBR***

- *Understanding the relationship between vegetation health and burn severity requires an understanding of the correlation between NBR and NDVI. Following a fire, the two indices typically have an inverse relationship: NDVI values decrease as vegetation density and health decline, while NBR tends to decrease in burned areas due to changes in vegetation structure and moisture. However, depending on variables like vegetation type, fire severity, and post-fire recovery procedures, the strength of this correlation may change.*
- *The correlation analysis shows how well the indices NBR, NDVI work for mapping forest fires and analyzing their effects. The findings show a strong linear relationship between changes in vegetation health and burn severity. This robust correlation demonstrates that these indices are very useful instruments for determining the effects of fires, evaluating the intensity of burns, and monitoring vegetation recovery, offering trustworthy information for forest fire planning and management.*

***Implications for Mapping Forest Fires***

- *The potential of NBR and NDVI for mapping forest fires is increased by their complementary nature. While NDVI's emphasis on vegetation health and density makes it especially helpful for tracking post-fire vegetation recovery, NBR's sensitivity to changes in moisture and vegetation structure makes it perfect for determining burn severity and burned areas. The use of these indices in conjunction with dNBR and dNDVI to map and evaluate the effects of fire is validated by the high correlation metrics.*

c) Detailed Findings: Expand on the findings related to the severity of fires, perhaps including more categories of severity or the ecological impact of different levels of burn severity

- *Thank you for the observation, regarding more levels/categories of burn severity. The findings from the remote sensing data reveal significant variations in fire severity and their ecological effects across the three forest reserves: Magamba FNR, Mkusu FR, and Shagayu FR. In both Magamba FNR and Shagayu FR, the majority of areas remained unchanged over the years, indicating minimal fire activity and stable vegetation conditions. This suggests that these ecosystems either have high resilience or are effectively managed, which helps in preserving habitats and maintaining soil stability. On the other hand, Mkusu FR displayed a marked shift in 2023, with high-severity burns and considerable high regrowth, indicating a major fire event that led to significant vegetation loss but was followed by a strong recovery. This highlights the ecosystem's ability to regenerate after disturbance.*
- *High-severity burns, such as those observed in Mkusu, lead to immediate ecological disruptions, including habitat loss and soil degradation. Despite this, the subsequent regrowth signifies a rapid ecological recovery, although it may alter species composition and competition dynamics over time. These findings emphasize the critical need for ongoing monitoring of burn severity levels to understand better their ecological impacts, guide restoration initiatives, and shape long-term fire management strategies, all of which are essential for sustaining forest resilience and ensuring sustainability.*
- *The* ***standard classes*** *of fire severity used for analysis include:*

*- High Severity: Areas heavily affected by fire, resulting in extensive vegetation loss and significant ecosystem disruption.*

*- Moderate-High Severity: Areas with noticeable vegetation loss but with some degree of ecological recovery potential.*

*- Moderate-Low Severity: Areas where fire effects are less intense, with partial vegetation loss and a quicker recovery.*

*- Low Severity: Areas with minimal fire impact, where the ecosystem remains largely intact.*

*- Unchanged: Areas that have not been significantly affected by fire over the study period.*

*- Low Regrowth: Areas with slow vegetation recovery following a fire event.*

*- High Regrowth: Areas exhibiting strong and rapid vegetation recovery after fire disturbance*

5. **Discussion**
a) Linking Results to Literature: Better integrate the results with existing literature, discussing how the findings align or contrast with other studies on forest fires in similar contexts.

- *Thank you, the manuscript has been revised to include more results that relate to existing literature. The section discusses how the findings align with or contrast with other studies on forest fires.*

b) Broader Implications: Discuss the broader implications of the findings for forest management and policy in Tanzania, including specific recommendations based on the results.

- *Further broader implications of the findings for forest fire management and policy in Tanzania, including specific recommendations based on the results have been revised in the manuscript.*

6. **Conclusion**
a) Future Research Directions: Suggest areas for future research that could build on the study’s findings, such as long-term ecological impacts of fires or the effectiveness of different fire management strategies.

- *Future research could focus on the long-term impacts of forest fires on biodiversity and ecosystems including on the effect on soil health, vegetation recovery patterns, and wildlife population dynamics in Western Usambara Mountain forests, and inform management practices*

b) Socio-economic Considerations: More deeply explore the socio-economic implications of the findings, particularly regarding local communities' dependence on forest resources and potential conflicts arising from fire management practices.

- *The study recommends enhancing community participation in forest conservation by increasing local knowledge and awareness of Community-Based Fire Management (CBFiM) and Integrated Fire Management (IFM) approaches. This involvement fosters a sense of ownership and shared responsibility, addressing encroachment, involvement in decision-making, and managerial challenges.*

c) Integration of Local Knowledge: Emphasize the importance of integrating local knowledge into fire management strategies, which could lead to more effective and culturally relevant solutions.

- *Integration of local knowledge can enhance trust between communities leaving adjacent the forest reserves and Government authorities, facilitating cooperation in implementing fire management initiatives that are culturally accepted and adhered.*

**Reviewer #2 Comments**

1. **Abstract**
a) Clarity and Conciseness: Ensure the abstract succinctly summarizes the study's objectives, methods, key findings, and significance. Avoid jargon and keep it accessible.

- *A well-revised abstract in all sections has been done as per the reviewer's contribution to the manuscript*

b) Results Highlight: Include specific quantitative results to give readers a clear idea of the study's impact.

- *Thank you, more quantitative results including percentages of the area affected by fire have been included in the manuscript.*

c) Key Terms: Consider defining critical terms (e.g., "burn severity") for clarity.

- *The definition of the key terms has been included and defined in the background section of the manuscript, following the reviewers’ suggestions.*

2. **Introduction**
a) Context and Rationale: Provide more background on the significance of forest fires in the Usambara Mountains. Why is this region particularly vulnerable?

- *The background of this study has been revised by including the significance of forest fires, as its unique biodiversity and complex ecosystem characterize the region, threatened by anthropogenic activities, Elevated mountainous forest areas and climate changes are some of the factors increasing its vulnerability*

b) Literature Review: Incorporate recent studies on forest fire mapping and severity assessment to highlight gaps your research addresses.

- *Recently studies have been incorporated in the revised manuscript to increase its clarity, highlighting critical gaps that the study addresses and issues on forest fire mapping and its severity assessment.*

c) Research Objectives: Clearly articulate the research questions or hypotheses guiding the study. Ensure these are explicitly linked to the issues raised in the introduction.

- *Thank you for the additional suggestion regarding linking the research questions with the issues discussed in the general background. The revised manuscript now incorporates this observation.*

3. **Methodology**
a) Geospatial Tools: Detail the specific geospatial tools and software used (e.g., remote sensing, GIS applications) and justify their selection.

- *Google Earth Engine (GEE) is a cloud-based satellite image processing platform that offers significant potential for forest fire mapping due to its high computational speed and ability to process large spatial datasets across broad areas. GEE allows users to efficiently analyze spatial data through codes and APIs, making it ideal for monitoring and assessing forest fires. The platform also supports data uploads, enabling users to integrate custom datasets with its extensive satellite imagery library, which is crucial for comprehensive fire analysis. GEE’s proven applications in land cover classification, forest, and vegetation monitoring, and ecosystem studies further enhance its suitability for tracking fire occurrences, impacts, and recovery. Its scalability and flexibility make it a powerful tool for large-scale forest fire mapping and monitoring efforts served as the platform for visualizing the map generated from GEE. QGIS, with its powerful geospatial analysis tools, enabled the creation of detailed visual representations of the study area. Additionally,* ***R Studio*** *was employed to plot time series graphs, providing a robust environment for statistical analysis and visualization of the data. The combination of GEE for data processing, QGIS for mapping, and R Studio for graphing offers a comprehensive approach to analyzing and visualizing forest fire dynamics and other related phenomena.*

b) Data Sources: Clarify the sources of data (e.g., satellite imagery, ground truth data) and their relevance to your study.

- *Landsat 8 and 9 imageries have significant potential for forest fire mapping due to the use of indices such as the Normalized Burn Ratio (NBR), Normalized Difference Vegetation Index (NDVI), delta NBR (dNBR), and delta NDVI (dNDVI). NBR is especially useful for detecting burn scars and assessing fire severity by comparing pre-and post-fire imagery, and Landsat's improved spectral bands improve its accuracy. The NDVI, which measures vegetation health, can help identify areas of vegetation loss during and after a fire, whereas dNBR provides a more detailed assessment of fire severity by calculating the difference in NBR before and after the fire. Similarly, dNDVI monitors vegetation changes and provides insights into post-fire vegetation recovery. Landsat 8 and 9 have high spatial and temporal resolution, which makes these indices powerful. Forest fire management and research greatly benefit from the high spatial and temporal resolution of Landsat 8 and 9, which make these indices effective tools for identifying burned areas, assessing the effects of fires, and tracking the regrowth of vegetation over time.*

c) Analysis Techniques: Provide more detail on the analytical methods (e.g., algorithms for assessing burn severity) and how they were implemented.

- *Forest fire mapping can benefit greatly from the use of Normalized Burn Ratio (NBR), Normalized Difference Vegetation Index (NDVI), delta NBR (dNBR), and delta NDVI (dNDVI), especially when processed and examined with Google Earth Engine (GEE). These indices offer supplementary data on areas affected by fire, the intensity of the burn, and the recovery of vegetation. The possible relationship between NBR and NDVI, as well as an explanation of how these indices can be used in GEE for mapping forest fires, are provided below:*
- *The Normalized Burn Ratio, or NBR, is a commonly used metric for determining the extent of fire and identifying burn scars, particularly in post-fire settings. It is especially vulnerable to variations in moisture content and vegetation, both of which are changed during a fire. The Near-Infrared (NIR) and Short-Wave Infrared (SWIR) bands in GEE can be used to compute NBR, which aids in the rapid identification of burned areas. A strong framework for tracking forest fires over wide spatial scales is provided by GEE's capacity to compute NBR over time and across large areas.*
- *Normalized Difference Vegetation Index (NDVI): This index is essential for evaluating the density and health of vegetation and can be used to monitor changes in vegetation both before and after a fire. Following a fire, a decline in NDVI values signifies the loss of vegetation and is useful in determining the size of areas affected by the fire. The long-term effects of forest fires can be better understood by using the time-series analysis of vegetation recovery that can be produced in GEE using NDVI.*
- *The delta Normalized Burn Ratio, or dNBR, provides a thorough analysis of the fire's severity by comparing the NBR values before and after the fire. Based on the extent of change, you can categorize areas into low, moderate, and high burn severity levels by computing dNBR in GEE. This is essential for forest fire management, rehabilitation, and recovery efforts as well as for gaining a better understanding of how the fire affected the landscape.*
- *dNDVI (delta Normalized Difference Vegetation Index): Like dNBR, dNDVI compares the NDVI values before and after a fire to determine the change in vegetation health. It sheds light on how severe vegetation loss and recovery are. The rate at which vegetation grows back after a fire can be tracked using dNDVI in GEE, which aids in tracking ecosystem recovery over time.*

***The relationship between NDVI and NBR***

- *Understanding the relationship between vegetation health and burn severity requires an understanding of the correlation between NBR and NDVI. Following a fire, the two indices typically have an inverse relationship: NDVI values decrease as vegetation density and health decline, while NBR tends to decrease in burned areas due to changes in vegetation structure and moisture. However, depending on variables like vegetation type, fire severity, and post-fire recovery procedures, the strength of this correlation may change.*
- *The correlation analysis shows how well the indices NBR, NDVI work for mapping forest fires and analyzing their effects. The findings show a strong linear relationship between changes in vegetation health and burn severity. This robust correlation demonstrates that these indices are very useful instruments for determining the effects of fires, evaluating the intensity of burns, and monitoring vegetation recovery, offering trustworthy information for forest fire planning and management.*

***Implications for Mapping Forest Fires***

- *The potential of NBR and NDVI for mapping forest fires is increased by their complementary nature. While NDVI's emphasis on vegetation health and density makes it especially helpful for tracking post-fire vegetation recovery, NBR's sensitivity to changes in moisture and vegetation structure makes it perfect for determining burn severity and burned areas. The use of these indices in conjunction with dNBR and dNDVI to map and evaluate the effects of fire is validated by the high correlation metrics.*

d) Limitations: Acknowledge any limitations of your methodology that could affect results (e.g., resolution of data, seasonal variations).

- *Thank you for this observation, generally using remote sensing for assessing burn severity has several limitations, including;*

i. *Remote sensing requires ground truthing to validate findings, but in some areas like in the Western Usambara Mountain Forest, it is very difficult to validate satellite observations since the areas are characterized by elevated mountains.*
ii. *Resolution limitation when capturing small or medium-sized fires which can either be under-estimated or over-estimated in the extent of burn areas and misclassify burn severity*
iii. *Both are effective when assessing areas shortly after the fire, but as vegetation begins to regenerate, the spectral signals change leading to potential misclassifications of burn severity, therefore distinguishing between burned and recovering areas becomes challenging.*
iv. *In the tropics both the NBR and NDVI are less effective due to quick changes in vegetation regrowth and quick changes in reflectance properties which can hinder the detection of fire impacts.*
4. **Results**
a) Presentation of Data: Use clear visuals (maps, graphs) to present findings effectively. Ensure figures are well-labeled and referenced in the text.

- *Thank you, Reviewer, for this observation; now the figures and tables included in the manuscript have been revised accordingly.*

b) Interpretation of Results: Offer insights into what the data reveals about fire severity and burned areas.

- *The revised manuscript now includes a more comprehensive interpretation of the results, linking fire severity with burned areas in terms of quantitative assessment, the spatial distribution of the affected area, and environmental factors such as topography in relation to fire occurrence.*

5. **Discussion**
a) Discuss any unexpected findings.

- *Thank you for this observation to be carefully included in the manuscript*

b) Statistical Analysis: If applicable, include details on statistical tests performed and their significance.

- *T Correlation metrics were used in this study statistical analysis to assess the relationships between the indices (NBR, NDVI, dNBR, and dNDVI). To evaluate the strength and predictability of these relationships, the correlation coefficient (R) and the coefficient of determination (R^2^) were computed. These metrics validated the use of these indices in mapping forest fires by offering important insights into the linear relationship between vegetation health and burn severity. The high correlation values highlight the importance and dependability of the indices in accurately capturing fire impacts and vegetation changes, even though no additional inferential statistical tests were carried out.*

6. **Conclusion.**
a) Summary of Findings: Recap the main findings succinctly, emphasizing their implications for forest management and policy.

- *Thank you for the observation, the revised manuscript has more details concerning the outcome of the findings, the implication on management aspects, and policy development*

b) Future Research: Suggest areas for future research based on your findings, particularly in relation to fire prevention and ecosystem recovery.
   - *Future research could focus on the long-term impacts of forest fires on biodiversity and ecosystems including on the effect on soil health, vegetation recovery patterns, and wildlife population dynamics in Western Usambara Mountain forests, and inform management practices*
c) Practical Applications: Discuss how this research could inform local conservation efforts or influence fire management strategies in the region

- *Thank you for more suggestions on how to improve the manuscript, in fact, the study informs local conservation on*

i. *Understanding fire dynamics through mapping fire severity and critical sources of forest fires such as agricultural activities adjacent to the forest reserves and inside the reserve, but also charcoal-making activities that can be solved by giving alternative sources of income like beekeeping activities and tree seedling to the community*
ii. *Biodiversity conservation and protection through identified critical burned areas and those areas with more risk to fire events and more efforts allocation for conservation through the clear decision making and,*
iii. *Community engagement by involving the community in conservation strategies, and measures by ensuring their needs and rights are observed*

**Overall comments**

i. Cohesiveness: Ensure each section flows logically into the next, maintaining a consistent narrative throughout the manuscript.

- *Thank you for the reminder, the revised manuscript has been kept in mind the scenario*

ii. Language and Style: Check for grammatical accuracy and stylistic consistency. Aim for a formal yet engaging tone.

- *Thank you again for this observation, we have gone through it at our level best*

iii. References: Ensure all cited works are up-to-date and relevant, following the appropriate formatting style.

- *The revised manuscript and cited works have been re-checked which are currently and relevant to the study.*

**Reviewer #3 Comments**

1. **Title**
a) On the cover page, the tittle of the manuscript deals about integrating geospatial tools in mapping forest fire severity and burned areas in the Western Usambara Mountain Forests. But, in the body of the manuscript, the locations of the study area were classified into different study village which may confuse the readers. So, tittle modifications should be required.

- *Thank you for this observation. To address potential confusion regarding the classification of study locations into different villages, the study area descriptions in the manuscript have been updated to align clearly with the title to ensure coherence between the title and the detailed study area discussions.*

2. **Abstract**
a) *Please elaborate the targeted model you used for data analysis for your study. On the line 15 and 16, the findings revealed that agricultural activities (44.5%) and charcoal production (21.1%) are the primary causes of these fires doesn’t explain the reality exist on fig.3a. you have to refer and edit it accordingly.*

- *Thank you for the observation in the abstract part and the area included in Fig 3a has been revised accordingly*
- *There was a typing error in this section but the main aim was to check on the main cause of the forest fires in the study area. Farm preparation contributed about 38.2% and not 44.5% as written in the abstract previously.*

3. **Introduction**
a) It is better if you add
   -The scientific definitions of forest fire
   -Lists of some native forests to the study area and a detail history about the forest fire before your study period in the study area.
   -Short and understandable research gap for your study.
   -Generalized forms of your study objective rather than the specific one.

- *Thank you for the inputs made regarding the manuscript, the scientific terms have been clarified, and the list of the native forests and their history have been revised as well as the objectives and the research gap of the study research.*

4. **Methodology**
a) Re-edit the geographical locations of the study area according the figure 1 reading.
   -Why don’t you specify your total study area? it was the mandatory to now the effects of forest fire on the total study area over the study period.
   -Please, specify the forest covers of the study area as the main description of the study area.

- *The geographical location of the study areas has been updated in Fig 1, the total study area and the total affected area by fire also have been added as well as the burn coverage in percentage. The forest cover of the study area has been also updated in the manuscript.*

b) Data sources
   -It was better if you put your geospatial data in tabular form with detail descriptions of data source, types, special resolution, specific purposes and downloaded year and time.

- *Thank you very much for this reminder, we have included these information in the main manuscript.*

-Specify the formula you used to limit your sample size for respondents’ nomination from household for interview.
-How many Focus Group Discussions (FGDs) you were organized during your study to summarize the whole idea raised from the respondents?

- *The procedure used for obtaining the household sample size in each village involved randomly by selecting 10% of household heads from the total number of households (N). The total number of households (N) in each village was divided by 10% to determine the sample size for that specific village. The number of households are presented in Table 2 as per Tanzania National Census of 2022 data. That is to say;*

$Sample size = N \times0.10$	*whereby N is the total number of households.*

- *We conducted six (6) Focus Group Discussions (FGD) as one of the tools of Participatory Rural Appraisal (PRA) during the data collection process, with each discussion consisting of 17 respondents from six villages. This approach facilitated the gathering of diverse perspectives and encouraged to rich discussions, allowing for a comprehensive understanding of the issues related to forest fires and the management strategies.*

5. **Results**
a) According to the results displayed on figure 4, the respondent’s perceptions and knowledge about forest fire were classified into six categories. So, what was your base for this classification? It was better if you increase and improve the visibility of the results presented on the figure 3a, b and 4 for the readers.

- *According to the results displayed in Fig.4, the respondent's perceptions and knowledge about forest fires were classified into six distinct categories. This classification was based on the researcher’s decisions during the interviews to focus on key areas of importance related to forest fire management. The topics were selected to ensure a comprehensive understanding of the community perspectives and experiences regarding the Forest Reserves protection and conservation from forest fires.*
- *The mentioned figures (3a, b and 4) have been revised in the manuscript to increase and improve readers visibility*

6. **Discussion and Recommendation**
a) Your discussion was organized and addressed the whole contents of your finding in comparison with pre conducted researches. However, your recommendation mainly for local community only. Are sure that local community alone can manage the effects of forest fire without involvements of governmental institutions?

- *Thank you for the appreciation. The recommendations have been reversed in the manuscript to cover both the social and remote sensing aspects with the involvement of the Government institution through various strategies and policies governing management*
